# Computational Analysis of Deleterious nsSNPs in *INS* Gene Associated with Permanent Neonatal Diabetes Mellitus

**DOI:** 10.3390/jpm14040425

**Published:** 2024-04-17

**Authors:** Elsadig Mohamed Ahmed, Mohamed E. Elangeeb, Khalid Mohamed Adam, Hytham Ahmed Abuagla, Abubakr Ali Elamin MohamedAhmed, Elshazali Widaa Ali, Elmoiz Idris Eltieb, Ali M. Edris, Hiba Mahgoub Ali Osman, Ebtehal Saleh Idris, Khalil A. A. Khalil

**Affiliations:** Department of Medical Laboratory Sciences, College of Applied Medical Sciences, University of Bisha, P.O. Box 551, Bisha 61922, Saudi Arabia; melnageeb@ub.edu.sa (M.E.E.); kmabdalla@ub.edu.sa (K.M.A.); aadlah@ub.edu.sa (H.A.A.); aaelamin@ub.edu.sa (A.A.E.M.); elshazali@ub.edu.sa (E.W.A.); elmoizie@ub.edu.sa (E.I.E.); aedris@ub.edu.sa (A.M.E.); hmosman@ub.edu.sa (H.M.A.O.); ebtihal@ub.edu.sa (E.S.I.); kaahmad@ub.edu.sa (K.A.A.K.)

**Keywords:** non-synonymous single nucleotide polymorphisms, insulin genes, permanent neonatal diabetes mellitus, genetic variations, computational methods, personalized medicine

## Abstract

*Insulin* gene mutations affect the structure of insulin and are considered a leading cause of neonatal diabetes and permanent neonatal diabetes mellitus PNDM. These mutations can affect the production and secretion of insulin, resulting in inadequate insulin levels and subsequent hyperglycemia. Early discovery or prediction of PNDM can aid in better management and treatment. The current study identified potential deleterious non-synonymous single nucleotide polymorphisms nsSNPs in the *INS* gene. The analysis of the nsSNPs in the *INS* gene was conducted using bioinformatics tools by implementing computational algorithms including SIFT, PolyPhen2, SNAP2, SNPs & GO, PhD-SNP, MutPred2, I-Mutant, MuPro, and HOPE tools to investigate the prediction of the potential association between nsSNPs in the *INS* gene and PNDM. Three mutations, C96Y, P52R, and C96R, were shown to potentially reduce the stability and function of the *INS* protein. These mutants were subjected to MDSs for structural analysis. Results suggested that these three potential pathogenic mutations may affect the stability and functionality of the insulin protein encoded by the *INS* gene. Therefore, these changes may influence the development of PNDM. Further researches are required to fully understand the various effects of mutations in the *INS* gene on insulin synthesis and function. These data can aid in genetic testing for PNDM to evaluate its risk and create treatment and prevention strategies in personalized medicine.

## 1. Introduction

Diabetes mellitus encompasses various metabolic disorders characterized by elevated blood sugar levels due to impaired insulin secretion or increased insulin resistance [1]. The dysfunction in the *INS* gene may contribute to the onset of diabetes mellitus [2]. The *INS* gene is located on chromosome 11 in humans. *Insulin* is the primary name for the gene [3].

The synthesis and production of insulin hormone take place in beta cells in the Langerhans islet of the pancreas [4,5]. These two processes begin with the synthesis of a longer precursor molecule called preproinsulin, the initial result of a translational product from the *INS* gene [2,4]. Preproinsulin is a 110-amino-acid polypeptide with a 24-amino-acid signal peptide. It is then cleaved to form proinsulin, which consists of an A chain, a B chain, and a connecting peptide (C-peptide). Proinsulin undergoes further cleavage to form mature insulin hormone and C-peptide. The C-peptide is released when insulin hormone is secreted and serves as a marker for its production [2,6]. The *INS* gene is transcribed into mRNA, which serves as a template for synthesizing insulin hormone. The mRNA is translated in the endoplasmic reticulum, and the nascent preproinsulin undergoes post-translational modifications, including signal peptide cleavage [5]. Insulin hormone is packaged into secretory granules within the pancreatic beta cells. It is released from beta cells in response to elevated blood glucose levels in the circulation to regulate and maintain the elevated blood glucose levels [7,8].

Neonatal diabetes typically manifests within the initial six months of life. Infants who experience the early onset of diabetes frequently possess mutations in one allele of the gene responsible for producing the insulin hormone [9]. Two types of neonatal diabetes mellitus have been reported: permanent neonatal diabetes mellitus (PNDM) and transient neonatal diabetes mellitus (TNDM) [10]. Mutations in the INS gene influence different phases of the biosynthesis of insulin hormone. These mutations are thought to interfere with either the cleavage process of the proinsulin chain or the assembly of the (A and B) chains to produce insulin, resulting in disturbed glucose levels in the blood [11,12]. *INS* gene mutations were described as a cause of PNDM [13,14].

As *INS* gene mutations were considered to affect the structure of insulin hormone and prescribed as a leading cause of PNDM, prediction of these mutations can lead to the early discovery or prediction of PNDM and better management and treatment of the disease. This study was conducted to identify potentially deleterious nsSNPs in the *INS* gene.

## 2. Methodology

### 2.1. Work Plan

**Figure 1:**
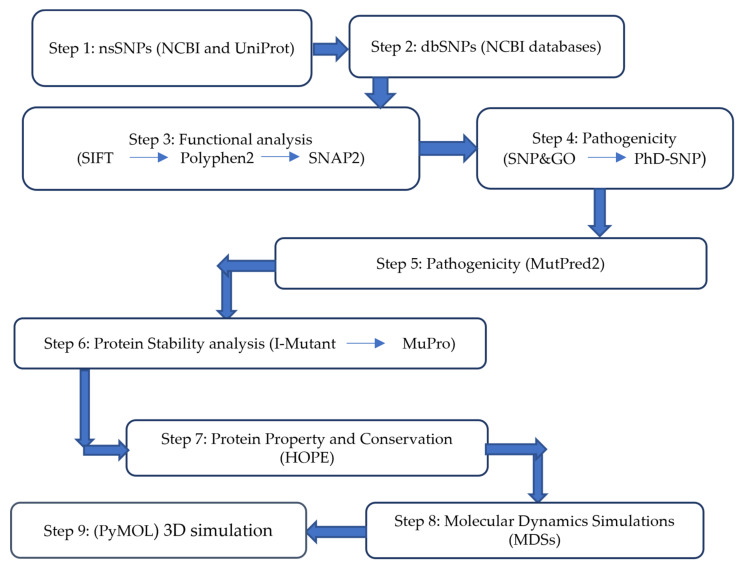
Flowchart to identify and categorize nsSNPs in the *INS* gene; each step indicates the tools used. If one nsSNP is considered deleterious in each step using certain tool it will be directly analyzed in the following tool or in the next step using another computational tool. In steps 8 and 9, tools are used to analyze and display structural changes.

This study utilized a computational approach consistent with other previous studies [15]. Different bioinformatics tools were used in this study to assess the deleterious effects of non-synonymous single-nucleotide polymorphisms (nsSNPs) in the *INS* gene. The nsSNPs considered to have high-risk profiles were analyzed to evaluate their stability, structural impact, and conservation. The methods used to identify and categorize putative functional nsSNPs in the *INS* gene are shown in Figure 1.

### 2.2. Data Collection

Data collection involved retrieving the nucleotide and amino acid sequences of the insulin protein, identified by accession numbers NP_001278826.1 and NG_050578.1, respectively, from the NCBI database in FASTA format. Information regarding single-nucleotide polymorphisms (SNPs) within the INS gene was obtained from the SNP database of NCBI via the following URL: http://www.ncbi.nlm.nih.gov/snp/ (accessed on 26 September 2023). Data of the *INS* gene and insulin protein were sourced from the PDB and UniProt databases, accessible using the web address https://www.uniprot.org/uniprotkb/P01308/entry (accessed on 26 September 2023).

### 2.3. Investigation of the Impact of nsSNPs on Protein Function

The impact of genetic variation on protein function was investigated using several tools, including Sorts Intolerant from Tolerant (SIFT), PolyPhen2, and SNAP2. The SIFT sequences were performed through the website https://sift.bii.a-star.edu.sg/www/SIFT_seq_submit2.html (accessed on 26 September 2023). SIFT predicts changes in protein function resulting from amino acid sequence alterations [16]. The website http://genetics.bwh.harvard.edu/pph2 (accessed on 26 September 2023) was accessed for PolyPhen2. The PolyPhen2 predicts the potential impact of amino acid substitutions on protein function and structure [17]. SNAP2 was accessed using https://www.rostlab.org/servces/snap/ (accessed on 26 September 2023). It predicts changes in secondary protein structure due to nsSNPs [18].

The SIFT, PolyPhen2, and SNAP2 tools were employed to assess the potential effects of SNPs; each offer unique approaches for classifying nsSNPs and determining confidence scores using various input criteria. These tools were employed to assess the functional consequences of non-synonymous single amino acid substitutions, differentiating between substitutions that are tolerable and those that are detrimental to protein function. SIFT predicts the impact of an amino acid change on protein function by analyzing sequence homology and the physical properties of amino acids [16]. A tolerance index score is assigned by SIFT, with scores below 0.05 indicating deleterious variations [16]. The PolyPhen2 assigns a numerical value ranging from 0 to 1, to forecast the potential effect of an amino acid substitution on the structure and function of a protein. A value of 0 indicates no impact, while a value of 1 indicates the most harmful consequence [17]. In contrast, SNAP2 conducts a comparative analysis of genomes to predict potential functional consequences of single amino acid substitutions at the protein level [18]. Deleterious SNPs identified through all mentioned servers underwent further computational analysis.

### 2.4. Prediction of SNP Disease Association

The correlation between filtered SNPs and diseases was assessed using SNPS & GO and Predictor of human Deleterious Single Nucleotide Polymorphisms (PhD-SNP) [19,20], accessible at http://snps-and-go.biocomp.unibo.it/snps-and-go/ (accessed on 26 September 2023) and http://snps.biofold.org/phd-snp/phd-snp.html (accessed on 26 September 2023), respectively. SNPS & GO utilizes Support Vector Machine (SVM) computational methods to detect harmful single amino acid substitutions, incorporating Gene Ontology (GO) annotations for classification. Variants scoring above 0.5 are deemed disease-causing [19]. The PhD-SNP utilizes a support vector machine (SVM) classifier to classify disease-associated SNPs. During the classification method, the categorization of amino acid changes as either neutral or disease-associated relies heavily on the exploitation of sequence and profile data [20]. The sequence profile calculation entails utilizing an input vector derived from the amino acid frequencies in both the wild-type and mutant sequences, the number of matched sequences, and the conservation score at the replacement site. If the PhD-SNP score exceeds 0.5, it indicates the existence of a mutation that is accountable for the onset of a disease [20]. The SNPs that were found to be responsible for causing disease and came across all mentioned servers were subjected to further computational analysis.

### 2.5. Prediction of SNPs on INS Protein Function Related to Pathogenicity

The MutPred2 web server, available at http://mutpred.mutdb.org (accessed on 26 September 2023), is used to classify mutations as either neutral or associated with disease [21,22]. This tool applies a machine-learning approach to predict the molecular mechanisms underlying the pathogenicity of amino acid substitutions. By analyzing a wide array of fifty different protein properties, it evaluates the effects of these substitutions. A score greater than 0.5 indicates a mutation that may be pathogenic [21].

### 2.6. Impact of SNPs on Protein Stability

The I-Mutant algorithm, leveraging Support Vector Machine (SVM) technology, forecasts changes in protein stability induced by missense nsSNPs [23]. Its output, known as the reliability index (RI), is scaled from 0 to 10, where a score of 10 represents the utmost reliability. I-Mutant is available at http://folding.biofold.org/i-mutant/imutant2.0.html (accessed on 26 September 2023). It requires the amino acid sequence of the INS protein and details about the mutating residues, such as their locations, for its analysis. Additionally, the MuPro tool, accessible at https://mupro.proteomics.ics.uci.edu (accessed on 26 September 2023), is employed for evaluating protein stability [24]. MuPro utilizes machine learning approaches, including SVM and Neural Networks, to predict how individual amino acid alterations affect protein stability. These methods were honed on an extensive database of mutations and validated through a 20-fold cross-validation process, achieving an accuracy rate of over 84%. Scores range between −1.0 and 1.0 to signify prediction reliability. A significant benefit of these approaches is their capacity to foresee alterations in protein stability without needing information on the tertiary structure.

### 2.7. Analysis of Protein Properties Utilizing the HOPE Tool

The HOPE software tool, which can be accessed at the URL https://www.cmbi.umcn.nl/hope (accessed on 27 September 2023), is designed to streamline the analysis of protein mutations [25]. It harnesses data from the UniProt database to assess the effects of single-nucleotide variations on the structural and functional aspects of proteins. HOPE produces in-depth reports that encompass textual explanations, graphical representations, and interactive visualizations, offering a detailed view of the mutation’s consequences. Mutants identified as deleterious nsSNPs by the previously mentioned tools were chosen for further scrutiny. These selected mutants underwent Molecular Dynamics Simulation (MDS) for an enhanced level of analysis.

### 2.8. Performing of Molecular Dynamics Simulations (MDSs)

To study changes in the structure over time in wild-type and mutant structures, Molecular Dynamics Simulations (MDSs) were carried out using GROMACS version 2020.6 on a Google Collaboration Pro notebook with substantial RAM. The GROMACS-OPLS-AA force field was used for initial calculations. Both systems were immersed in cubic boxes filled halfway with water molecules, with a minimal radius of 1 nm. To balance the system, 10 sodium ions (Na^+^) were introduced using the GROMACS genion tool. Energy minimization was achieved through the steepest descent algorithm, with an energy step size of 0.01 and a maximum of 50,000 iterations. The system’s stability was preserved at a Parrinello–Rahman pressure of 1 bar and a Berendsen temperature of 300 K. Electrostatic interactions were computed using the Partial Mesh Ewald (PME) technique, with a short-range cut-off for electrostatic and van der Waals interactions set at 1.0 nm. The neighbor list was updated every 10 ps. The LINCS algorithm, with a time step of 0.002 ps, maintained all bond constraints, including heavy atom-H bonds. An isothermal compressibility of 4.5 × 10^−5^ was used, with coupling constants for temperature and pressure set to 0.1 ps and 2.0 ps, respectively. The system underwent equilibration for 100 ps in both the NPT (constant number of particles, pressure, and temperature) and NVT (constant number of particles, volume, and temperature) ensembles, with a Berendsen temperature of 300 K and a Parrinello–Rahman pressure of 1 bar. Trajectories were recorded every 1 ps during 10 ns of molecular dynamics simulations for both wild-type and mutant structures. Various analysis tools like g_rms, g_rmsf, g_sasa, g_Rg, and g_density were utilized to assess parameters such as the RMSD (root-mean-square deviation), RMSF (root-mean-square fluctuation), SASA (solvent accessible surface area), and Rg (radius of gyration) to compare structural differences between the wild-type and mutant structures.

### 2.9. Displaying Three-Dimensional Structural Change Using PyMol Software

A 3D simulation of the wild-type INS protein and putative mutations was performed to display structural changes using the PyMol software Version 2.0.

## 3. Results

This study aimed to identify the potential deleterious nsSNPs in the *INS* gene by implementing computational algorithms including SIFT, PolyPhen2, SNAP2, SNPs & GO, PhD-SNP, MutPred2, MuPro, and I-Mutant. These algorithms analyze the influence of nsSNPs on *Insulin* protein function, stability, and disease association. Subsequently, the HOPE software tool was utilized to evaluate the effect of nsSNPs on the structural and functional properties of the *INS* gene. The nsSNPs predicted by all utilized algorithms were analyzed for structural changes over time, in both wild-type and mutant structures, through MDSs using the GROMACS tool.

### 3.1. Data Collection

SNPs associated with the *INS* gene and its corresponding *Insulin* protein sequence, identified by UniProt ID P01308, were obtained from the NCBI dbSNP and UniProt Knowledgebase databases. A total of 8,147,365 SNPs were mapped to the *INS* gene sequence. Subsequently, 130 nsSNPs were detected in the coding region. These nsSNPs are suspected to have the potential to cause missense mutations and significantly impact protein structure and function. The nsSNPs present in the coding sequence of the *INS* protein were considered for our analysis.

### 3.2. Exploring the Influence of SNPs on Protein Function

The analysis in this study focused on predicting nsSNPs deleteriousness on the *INS* gene to determine their potential impact on structural and functional properties. Out of 130 nsSNPs, 86 were classified as deleterious by the SIFT tool. Additionally, 67 nsSNPs were identified as probably damaging and potentially harmful by the PolyPhen2 tool. Confirmation of the harmful effect of these nsSNPs was conducted using SNAP2. All identified nsSNPs using the PolyPhen2 tool were confirmed as deleterious by SNAP2. Thus, a total of 67 nsSNPs were identified as the most deleterious based on the above-mentioned software (Table 1). These 67 nsSNPs were used for further analysis in the next predicting tools.

### 3.3. Prediction of SNP–Disease Association

A combined approach utilizing the SNP & GO and PhD-SNP tools was used to analyze the 67 nsSNPs for disease susceptibility. Results of SNP & GO suggested that all 67 nsSNPs were considered to have the potential to be associated with disease, with scores ranging from 9 to 10. However, PhD-SNP analysis identified only 12 nsSNPs out of the 67 as significantly correlated with disease. The remaining 55 nsSNPs showed neutral effects (Table 2). These 12 variants were subjected to additional analysis to obtain their potential impact.

### 3.4. Prediction of SNPs on INS Protein Function Related to Pathogenicity

This study examines the genetic pathways that cause disease and presents the probability ratings obtained from analyzing 12 different nsSNPs using the MutPred2 server. MutPred2 analysis categorized 8 out of the 12 examined nsSNPs as variants associated with pathological conditions, indicating alterations in the protein’s structure (Table 3). Changes included a loss of helix, disulfide linkage, and loop, and altered transmembrane protein, signal peptide, ordered interface, and metal binding. However, MutPred2 predicted a neutral impact on protein function for four mutants.

### 3.5. Predicting the Impact of SNPs on Protein Stability

I-Mutant2.0 and MuPro software were used to investigate the impact of predicted deleterious mutations on *INS* protein stability. Findings derived from I-Mutant2.0 exhibited coherence, indicating that seven nsSNPs were anticipated to diminish the protein’s stability in a decreasing manner. However, only one nsSNP was found to increase protein stability. However, findings derived from MuPro indicated that four nsSNPs out of the seven were anticipated to diminish the protein’s stability. Three nsSNPs were found to increase protein stability (Table 4).

### 3.6. Analysis of Protein Properties

The HOPE tool analysis revealed that three nsSNPs were predicted to damage protein structure. However, L14Q was found not to affect the protein structure (Table 5). From the HOPE report, the mutations (P52R) Proline into Arginine at position 52, (C96Y) Cysteine into Tyrosine at position 96, and (C96R) Cysteine into Arginine at position 96 showed that the mutant residue was bigger than the wild-type residue in all mutations. And the Wild-type residue was more hydrophobic than the mutant residue. However, the Wild-type residue charge was NEUTRAL and the Mutant residue charge was POSITIVE in mutations (P52R and C96R), with no charge change in mutation (C96Y). The Mutant and the wild-type residues were not very similar in all mutants (P52R, C96Y, and C96R). All three mutations were considered probably damaging to the protein.

### 3.7. Molecular Dynamics Simulation (MDS) Results

**Figure 2:**
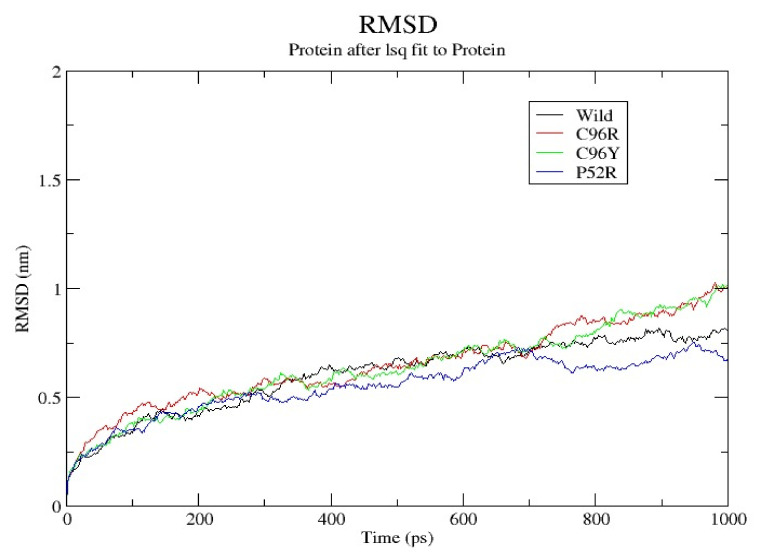
A Root-Mean-Square Deviation (RMSD) analysis of proteins throughout molecular dynamics (MD) simulations.

Figure 2 displays a Root-Mean-Square Deviation (RMSD) analysis of proteins throughout molecular dynamics simulations (MDSs). The RMSD graphic demonstrates the conformational stability of a wild-type protein in comparison to its three variations, C96R, C96Y, and P52R, throughout a 1000 ps simulation period. Each variety is represented by a distinct colored line, whereas the wild type is displayed in black. The path of the wild-type protein is used as a standard to evaluate how mutations affect protein stability. Upon initial examination, all protein variations, including the wild-type, exhibited a rise in RMSD values as the simulation advances, a common occurrence as the protein navigates through different conformations. The wild-type stabilized at about 0.6 nm, showing a plateau after initial fluctuations. The C96R mutation, highlighted in red, showed a comparable level of stabilization, indicating that this mutation does not greatly affect the protein’s structural stability. The C96Y variant, shown in green, displayed a slightly greater RMSD, reaching approximately 0.7 nm, suggesting a potential minor increase in structural flexibility or a departure from the original structure. The P52R mutation, highlighted in blue, exhibited a consistent rise in RMSD, peaking at about 1.5nm, indicating significant structural alterations or instability caused by this mutation. The RMSD study shows that the P52 R mutation has a large effect on the protein’s structural stability, whereas the C96R mutation has a minimal influence, and the C96Y mutation causes a minor increase in flexibility. These results suggest that the mutation at position 52 causes a greater departure from the protein’s original structure compared to mutations at position 96. None of the variants exhibited symptoms of reaching a plateau within the 1000 ps duration. The wild-type protein remained stable for around 200 ps, serving as a reference point for studying the characteristics of its many forms. The RMSD analysis data are crucial for comprehending the structural consequences of these mutations and their possible influence on the protein’s function.

**Figure 3:**
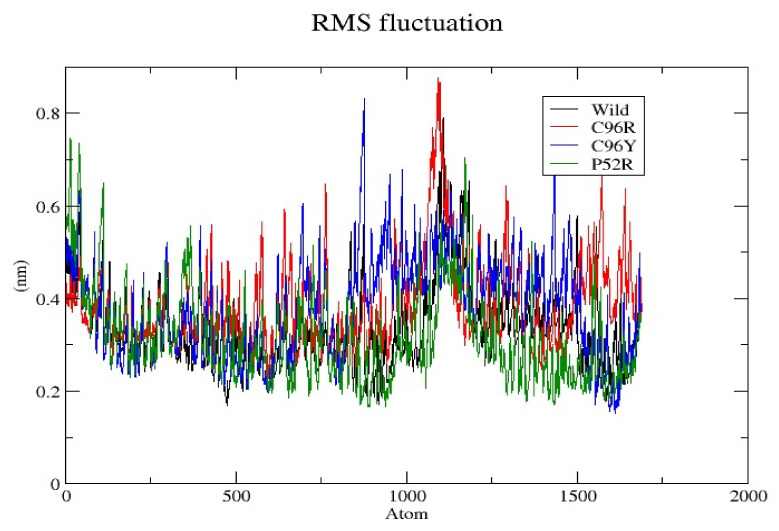
The Root-Mean-Square fluctuation (RMSF) of individual atoms in a protein throughout the MD simulation.

Concerning the results presented in Figure 3, the graph displays the Root-Mean-Square fluctuation (RMSF) of individual atoms in a protein throughout the MD simulation. The RMSF quantifies the degree of atomic movement from their mean position, indicating the variability in flexibility across various regions of the protein structure. The RMSF graph illustrates the flexibility of a wild-type protein and its variations, C96R, C96Y, and P52R, spanning approximately 2000 atoms. A line graph illustrates the flexibility of each protein variant, with distinct colors representing each variant, including the wild-type one. Fluctuations are quantified in nanometers (nm), with the vertical axis indicating the extent of fluctuation and the horizontal axis indicating the atom’s position in the protein. Upon analysis, it is clear that the wild-type protein displayed a fluctuation pattern that acts as a reference for comparing with the mutations. All variations exhibited regions with high and low fluctuation, indicating sections of the protein that are either more flexible or stiffer. The C96R variant (in red) typically aligned with the behavior of the wild type, indicating that the mutation does not significantly alter the protein’s flexibility. The C96Y variant (in green) and P52R variant (in blue) exhibited higher fluctuation in certain places, suggesting probable areas of enhanced flexibility or disorder perhaps caused by the mutations. The P52R variant displayed prominent peaks, indicating potential structural changes or increased mobility in certain protein areas relative to the wild-type and other variants. The data show that the P52R mutation caused significant variation in atomic locations, which could impact the structural integrity and function of the protein. The C96Y mutation enhanced flexibility to a lesser extent than P52R. The C96R mutation had little effect on flexibility, closely approaching the behavior of the wild-type protein. Fluctuations in RMSF are important for comprehending the functional impacts of mutations. Areas with increased fluctuations may indicate active or binding sites, or structural domains crucial for protein stability.

**Figure 4:**
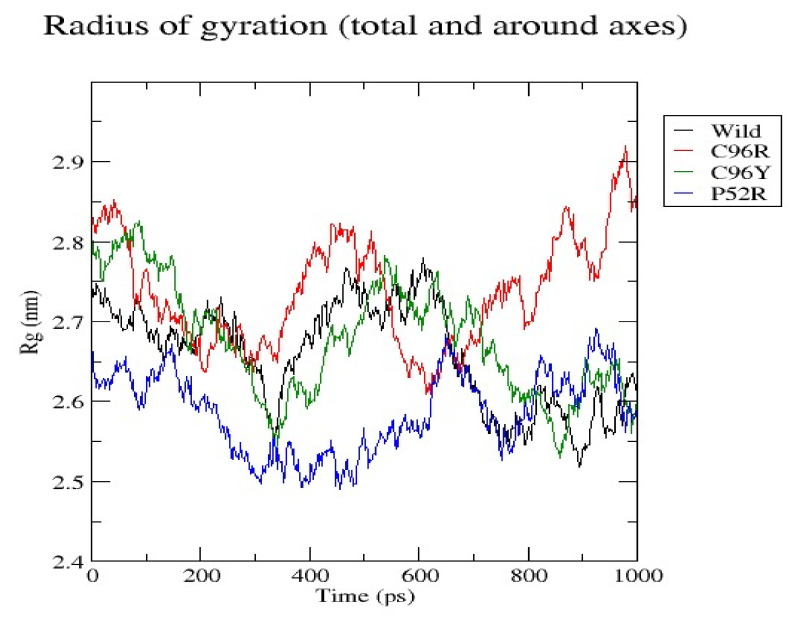
The radius of gyration of a wild-type protein and its three variations (C96R, C96Y, and P52R) as a function of time throughout a simulation duration of 1000 ps.

In Figure 4, the radius of gyration (Rg) of a wild-type protein and its three variations, C96R, C96Y, and P52R, as a function of time throughout a simulation duration of 1000 ps is shown. The Rg quantifies the compactness of a protein and the distribution of its mass relative to an axis. The figure illustrates the difference in compactness between the wild-type protein and its variations during the MD simulation. The radius is quantified in nanometers (nm) and monitors alterations in the protein’s morphology and dimensions over time. The graph displays the average distance of the protein’s atoms from its center of mass, with each line representing a different version indicated by different colors. A larger radius of gyration indicates a less compact protein structure, whereas a smaller number suggests a more compact structure.

During the simulation, all protein structures displayed variations in their radius of gyration, suggesting alterations in compactness and tertiary structure as time progressed. The wild-type protein exhibited varying levels of compactness, with the radius of gyration values oscillating between around 2.5 nm and 2.9 nm. The C96R variation (red line) displayed comparable fluctuations, indicating that this mutation does not substantially change the overall compactness of the protein. The C96Y variant (green line) showed a somewhat larger radius of gyration across most of the simulation, suggesting a less dense structure compared to the wild type. The P52R variant showed a unique pattern where its radius of gyration stayed consistently lower than the others during the initial half of the simulation but eventually increased to reach the highest level of extension. This indicates a notable structural change caused by the mutation. The data from the radius of gyration research indicate that the P52R mutation has the most significant impact on the protein’s compactness, potentially influencing its stability and function. The C96Y mutation slightly reduced the protein’s compactness; however, the C96R mutation had less impact on the protein’s compactness relative to the wild type. The differences in the radius of gyration, especially in the P52R variant, may indicate modified functional dynamics or alterations in the protein’s capacity to interact with other molecules. These findings can impact the comprehension of the structural and functional characteristics of the protein being analyzed.

**Figure 5:**
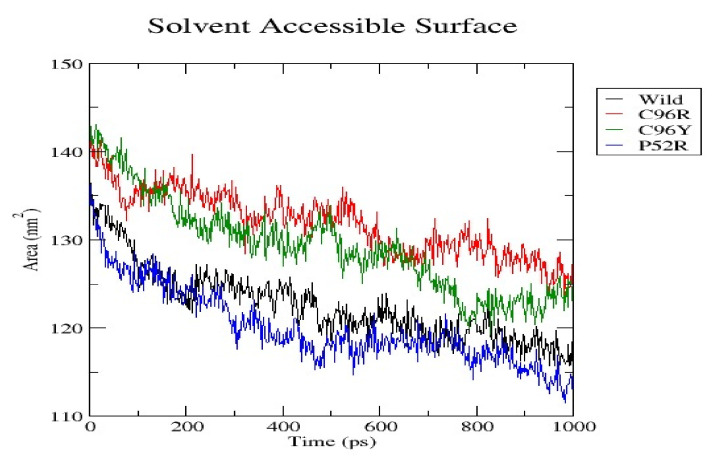
The Solvent Accessible Surface Area (SASA) of a wild-type protein and the three variants (C96R, C96Y, and P52R) during a 1000 ps molecular dynamics simulation.

Figure 5 shows the Solvent Accessible Surface Area (SASA) of a wild-type protein and three variations, C96R, C96Y, and P52R, during a 1000 ps molecular dynamics simulation. SASA is a crucial structural metric that represents the surface area of a protein available for interaction with a solvent, usually water. This parameter is pertinent to the stability, folding, and interactions of proteins. The SASA figure displays how the surface area exposed to solvent changed over time for both the wild-type and mutant proteins during the simulation. Values are expressed in square nanometers (nm^2^), with time shown in ps. The wild-type protein is shown by the black line, and the mutants are illustrated in red (C96R), green (C96Y), and blue (P52R). Initial observations demonstrate that all protein variations exhibited a reduction in SASA over time during the simulation, indicating a possible shift towards a more condensed structure or a modification in conformation dynamics, leading to decreased solvent exposure. The C96R variant initially exhibited SASA similar to the wild type but deviated towards the end, suggesting minor variations in exposure or folding patterns that are absent in the wild type. Throughout the simulation, the C96Y and P52R variations consistently displayed higher SASA values than the wild type, with the C96Y variant displaying the most notable difference. This may suggest a more open conformation or changed folding pattern that enhances the solvent accessibility of specific residues. The P52R variation had higher SASA values than the wild type and displayed a complicated pattern with frequent fluctuations, indicating a potentially more dynamic or flexible protein structure. This study indicates that mutations at positions 96 and 52 impact the protein’s interaction with the solvent, potentially influencing protein stability and function. The heightened solvent exposure in the C96Y and P52R variations could impact the protein’s surface chemistry and its ability to interact with other biomolecules. The reduced SASA observed in the later stages of the simulation for the C96R variant compared to the wild type may suggest changes in the tertiary structure or dynamics that could impact the protein’s biological function. The results emphasize the significance of SASA in elucidating the impact of mutations on protein structure and function.

**Figure 6:**
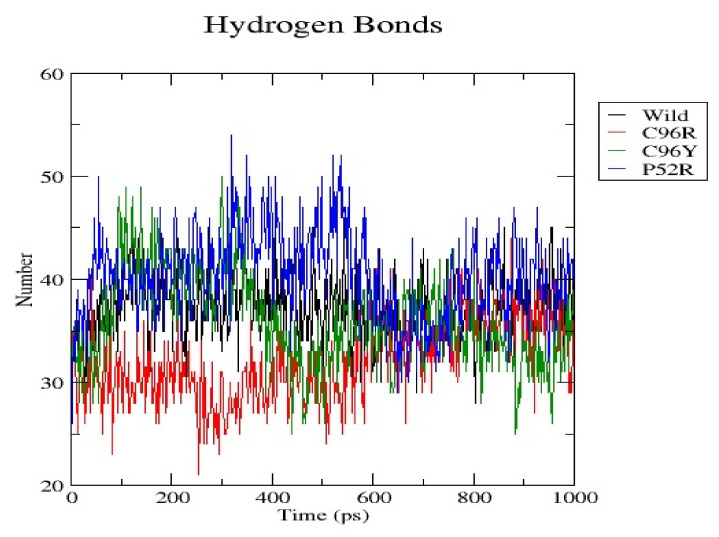
Quantification of the hydrogen bonds present in a wild-type protein and its three variations (C96R, C96Y, and P52R) over a 1000 ps molecular dynamics simulation.

Figure 6 quantifies the hydrogen bonds present in a wild-type protein and its three variations, C96R, C96Y, and P52R, over a 1000 ps molecular dynamics simulation. Hydrogen bonds play a crucial role in maintaining the stability of protein secondary and tertiary structures and can impact protein dynamics. The graph measures the hydrogen bonding arrangements in the molecular structures of the wild-type and mutant proteins as time progresses. The number of hydrogen bonds in the wild-type protein is represented by a black line that varies during the simulation, demonstrating the dynamic nature of intramolecular interactions. The mutations C96R, C96Y, and P52R are depicted by red, green, and blue lines, respectively, each displaying a unique hydrogen bonding arrangement in comparison to the original type. The comparison of hydrogen bond dynamics shows that the wild-type protein typically maintained a specific range of hydrogen bonds, mostly between 35 and 55. The C96R variant closely resembled the wild type in the amount of hydrogen bonds, indicating that this mutation does not significantly change the hydrogen bonding network. The C96Y variation had a wider range and a slightly larger quantity of hydrogen bonds, suggesting potential changes in intramolecular interactions or the enhanced stability of the secondary structure. The P52R variation exhibited the highest variety in hydrogen bond numbers, potentially indicating alterations in the protein’s structural stability and folding pattern as a result of the mutation. Proteins’ structural integrity and function are frequently dependent on their hydrogen bonding networks. The data indicate that the P52R mutation had the most significant impact on hydrogen bond formation during the simulation, potentially leading to substantial changes in protein structure and dynamics. The C96Y mutation exhibited enhanced hydrogen bonding but did not display the same level of variability as the P52R mutation, indicating a less significant effect on the protein’s stability. The similarity in hydrogen bond patterns between the C96R variation and the wild type suggests that this mutation has a limited impact on the protein’s structural stability. The observations could impact the protein’s heat stability, folding dynamics, and interaction with other molecules.

### 3.8. Displaying Three-Dimensional Structural Changes Using PyMol Software

**Figure 7:**
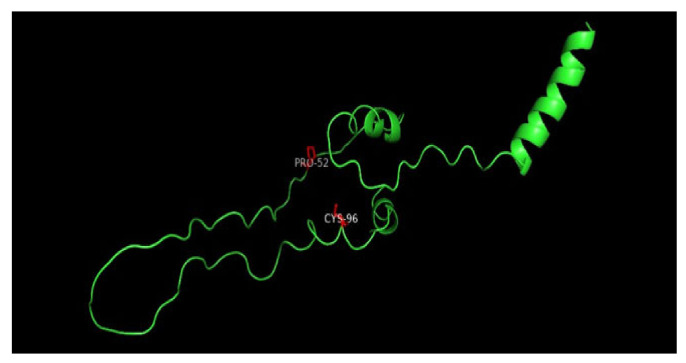
Three-dimensional structure of the wild-type INS protein (PDB ID: 4i30), and its mutations, P52R, C96R, and C96Y. The sites of the mutations are shown in the surface presentation and red color. This figure was prepared using PyMol Version 2.0.

**Figure 8:**
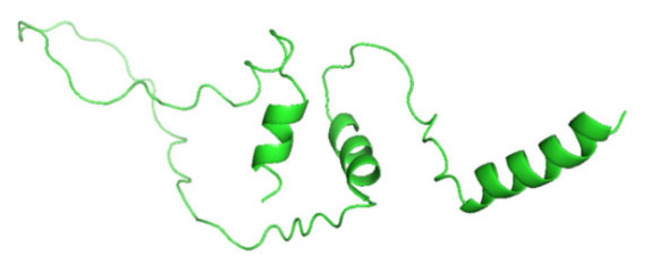
The three-dimensional structure of the wild-type INS protein (PDB ID: 3i40). This figure was prepared using PyMol software.

**Figure 9:**
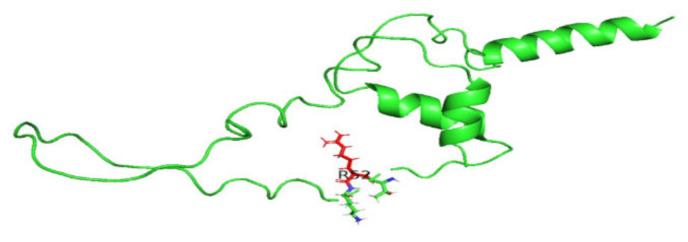
The three-dimensional structure of the INS protein (PDB ID: 3i40) for the mutation P52R; the site of the mutation is red. This figure was prepared using PyMol software.

**Figure 10:**
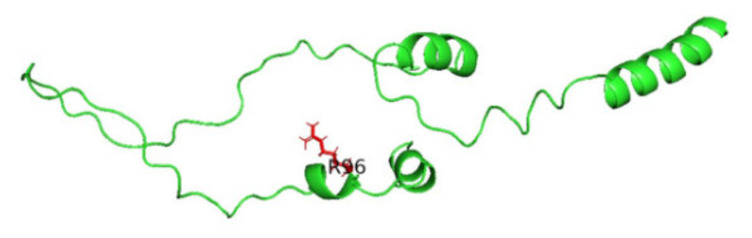
The three-dimensional structure of the INS protein (PDB ID: 3i40) for the mutation C96R; the site of the mutation is red. This figure was prepared using PyMol software.

**Figure 11:**
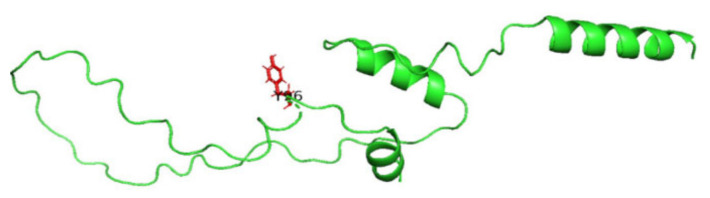
The three-dimensional structure of the INS protein (PDB ID: 3i40) for the mutation C96Y; the site of the mutation is red. This figure was prepared using PyMol software.

Figure 7 displays the three-dimensional structure of the INS protein (PDB ID: 3i40), the wild type, and its mutations P52R, C96R, and C96Y. The sites of the mutations are shown in the red color. Figure 8, Figure 9, Figure 10 and Figure 11 display the three-dimensional structure of the wild type and its mutations, P52R, C96R, and C96Y, respectively.

## 4. Discussion

In this study, we investigated the impact of nsSNPs in the *INS* gene on PNDM using computational methods. In this research, we sought to enhance the understanding of genetic factors influencing PNDM and their potential implications for personalized medicine. Our findings are expected to highlight the molecular basis of the illness, identify new diagnostic indicators, and uncover potential treatment targets. This study aligns with research that identified the association of nsSNPs with diabetes mellitus, and research emphasized the relevance of genetic changes in the *INS* gene in the development of diabetes mellitus [26].

Many computational methods were used to analyze the impact of nsSNPs on the *INS* gene protein structure and function that are potentially contributing to the onset of PNDM. The approach outlined in this study for predicting the deleteriousness of nsSNPs within the *INS* gene is comprehensive and systematic, utilizing multiple tools to assess the potential impact on protein structure and function. The use of SIFT, PolyPhen2, and SNAP2 tools provides a multifaceted analysis, enhancing confidence in identifying harmful variants (Table 1). Comparing our study with other similar research using computational tools can provide insights into the consistency and reliability of our findings. A study by Kumar et al. (2009) conducted a comprehensive evaluation of the SIFT and PolyPhen2 algorithms for predicting the impact of amino acid substitutions on protein function. Kumar et al. (2009) found that while both tools generally perform well, there can be discrepancies in their predictions, highlighting the importance of using multiple prediction methods for increased accuracy [27]. Furthermore, the incorporation of SNAP2 as a confirmation tool adds another layer of validation to the predictions made by PolyPhen2, enhancing the robustness of the findings. Additionally, a study performed by Ioannidis et al. (2016) emphasized the need for caution when interpreting computational predictions of nsSNP deleteriousness, highlighting the potential for false positives and false negatives. They underscored the importance of experimental validation to confirm the functional impact of predicted variants [28]. Our study employed a rigorous approach to predict the deleteriousness of nsSNPs within the *INS* gene, incorporating multiple computational tools and validation steps. This approach enhanced confidence in the identified deleterious variants.

A combined approach was utilized using SNP & GO and PhD-SNP tools to analyze the disease susceptibility of nsSNPs within the *INS* gene to provide valuable insights into their potential association with diseases. SNP & GO analysis provides a score range from 9 to 10, suggesting a potential association of 67 nsSNPs with the disease (Table 2). This method likely considers functional annotations and gene ontology terms associated with the SNPs to infer their potential disease relevance. On the other hand, the PhD-SNP methodology identifies only 12 out of the 67 nsSNPs as significantly correlated with disease, with the remaining 55 nsSNPs predicted to have neutral effects (Table 2). A study conducted by Martelotto et al. (2014) evaluated the performance of SNP & GO in predicting the disease association of nsSNPs. They found that while SNP & GO can provide valuable insights into the potential functional impact of nsSNPs, it may also yield false positive predictions due to the reliance on gene ontology annotations, which can be incomplete or inaccurate [29]. In contrast, the PhD-SNP methodology employs a different approach, likely incorporating features such as protein structure, evolutionary conservation, and functional annotations to predict disease association. A study conducted by Bendl et al. (2014) demonstrated the effectiveness of PhD-SNP in identifying disease-associated nsSNPs, highlighting its utility in prioritizing variants for further analysis [30]. The identification of 12 nsSNPs as significantly correlated with disease by PhD-SNP suggests a subset of variants with a higher likelihood of impacting disease susceptibility. While SNP & GO and PhD-SNP provide valuable insights into the potential disease association of nsSNPs, the differences in their predictions highlight the need for the careful consideration and validation of computational findings.

Then, MutPred2 was applied to analyze nsSNPs within the *INS* gene to provide valuable insights into nsSNPs’ potential impact on protein functionality related to pathogenicity. The identification of eight nsSNPs associated with pathological conditions suggests a subset of variants with a higher likelihood of disrupting normal protein function (Table 3). The specific alterations predicted by MutPred2, such as the loss of helix, disulfide linkage, loop, transmembrane protein, signal peptide, ordered interface, and metal binding, provide mechanistic insights into how these nsSNPs may lead to pathogenicity. Comparing these results with findings from other analyses, such as results obtained from SIFT, PolyPhen2, SNAP2, SNP & GO, and PhD-SNP, can help corroborate and contextualize the predictions made by MutPred2. For instance, if an nsSNP is predicted to be deleterious by multiple algorithms and also associated with disease by SNP & GO and PhD-SNP, it strengthens the confidence in its potential pathogenicity, as indicated by MutPred2.

A study by Pejaver et al. (2017) evaluated the performance of MutPred2 in predicting the pathogenicity of nsSNPs and found it to be one of the top-performing tools in terms of sensitivity and specificity. However, they also emphasized the importance of integrating predictions from multiple tools and experimental validation to improve the accuracy of pathogenicity predictions [21]. The MutPred2 analysis provides valuable insights into the potential impact of nsSNPs on *INS* protein functionality related to pathogenicity [21].

Assessment of predicted deleterious mutations on *INS* protein stability using both I-Mutant2.0 and MuPro tools provides valuable insights into the potential impact of nsSNPs on protein stability. However, the discrepancies between the predictions obtained from these two tools warrant careful consideration and comparison with findings from the other analyses. In this study, results from I-Mutant2.0 suggested that seven out of eight of the analyzed nsSNPs are expected to diminish protein stability, with only one nsSNP predicted to increase stability. Conversely, the findings from MuPro indicated that four out of seven nsSNPs are anticipated to diminish protein stability, while three are predicted to increase stability (Table 4). Comparing these results with findings from other computational tools, such as SIFT, PolyPhen2, SNAP2, and MutPred2, can help assess the consistency of predictions regarding the impact of nsSNPs on protein stability. If an nsSNP is consistently predicted to be deleterious by multiple tools and associated with disease susceptibility, it strengthens the confidence in its potential to disrupt protein stability and function.

Khan, et al. (2010) evaluated the performance of I-Mutant2.0 and MuPro in predicting the impact of nsSNPs on protein stability. They found that while these tools can provide useful predictions, there can be discrepancies between their results due to differences in underlying algorithms and features considered. While the predictions from I-Mutant2.0 and MuPro provide insights into the potential impact of nsSNPs on *INS* protein stability, discrepancies between the results highlight the importance of considering multiple computational tools [31].

The use of the HOPE tool to evaluate the effects of single-nucleotide variations (SNVs) on the structural and functional properties of proteins, specifically focusing on the four nsSNPs predicted to diminish protein stability by the MuPro tool, provides additional insights into their potential impact. The identification of three nsSNPs (C96Y, P52R, and C96R) as potentially damaging to the protein’s structure by the HOPE tool aligns with findings from other computational analyses, such as MutPred2, which predicted alterations in protein structure for these variants. This consistency across multiple prediction methods strengthens the confidence in the detrimental effects of these nsSNPs on protein structure and function. Conversely, our study finding that the L14Q variant was not predicted to affect protein structure by the HOPE tool (Table 5) contrasts with its classification as deleterious by other computational tools, such as MutPred2, I-Mutant, and MuPro. This discrepancy highlights the importance of considering multiple prediction methods and experimental validation to accurately assess the impact of nsSNPs on protein structure and function.

Venselaar et al. (2010) evaluated the performance of the HOPE tool in predicting the effects of mutations on protein structure and function. They found that HOPE can aid in understanding the potential impact of gene mutations on protein stability and function [25]. Therefore, using the HOPE tool to evaluate the effects of nsSNPs on protein structure and function complements findings from other computational analyses.

Our investigations pinpointed four variations as the most harmful using the combined results of different analytical methods. Three possible mutants, named C96Y, P52R, and C96R, were chosen among the four nsSNPs, identified using the HOPE tool (Table 5) as the most harmful mutants, for further study using molecular dynamic simulations (MDSs). The detected mutations were shown to alter the protein’s stability and functional characteristics, potentially affecting its biological activity. A molecular dynamics simulation for these three nsSNPs was conducted to assess the differences in protein dynamics between the wild-type and mutant *INS* proteins (Figure 2, Figure 3, Figure 4, Figure 5 and Figure 6).

In the analysis of nsSNPs in the *INS* gene, molecular dynamics simulations can be employed to understand the structural dynamics and stability of the *INS* protein affected by the variants.

RMSD analysis evaluated the overall structural stability of proteins during the MD simulations. Our findings suggest that while the C96R mutation has minimal impact on stability, the C96Y mutation causes a slight increase in flexibility, and the P52R mutation induces significant structural alterations, as evidenced by a higher RMSD value. These observations align with computational predictions and highlight the structural consequences of these mutations. RMSF analysis provides insights into the flexibility of individual atoms within the protein structure. Results indicate that the C96R mutation closely resembles the wild type, while the C96Y and P52R mutations exhibit increased fluctuations in certain regions, suggesting potential alterations in flexibility and structural dynamics caused by these mutations. The radius of gyration reflects the compactness of the protein structure. Data showed that while the C96R mutation has minimal impact on compactness, the C96Y mutation slightly reduces it, and the P52R mutation significantly increases it, indicating substantial structural changes induced by this mutation. SASA analysis revealed changes in the protein’s interaction with the surrounding solvent. Results suggest that the C96Y and P52R mutations increase solvent accessibility, potentially affecting surface chemistry and molecular interactions, whereas the C96R mutation has a limited impact on solvent exposure. Hydrogen bonds are crucial for maintaining protein stability and structure. Data indicate that while the C96R mutation maintains a similar hydrogen bonding pattern to the wild type, the C96Y mutation exhibits enhanced hydrogen bonding, and the P52R mutation displays variability, suggesting significant alterations in protein stability and folding dynamics.

These computational analyses provide valuable insights into the structural and functional consequences of specific mutations in the insulin protein. The detected mutations were shown to alter the protein’s stability and function, potentially affecting its biological activity. A molecular dynamics simulation was conducted to assess the differences in protein dynamics between the wild-type and mutant *INS* proteins. The simulation lasted 1000 picoseconds and involved analyzing various metrics such as the RMSD, RMSF, SASA, and RG. Comparisons were conducted to analyze the functional traits and robustness of the protein structures. After minimizing, marginal variances in the RMSD plots of backbone atoms showed that the wild-type structure was more stable than the mutations. The RMSF plots showed that the native and mutant structures displayed the highest degree of flexibility. The RMSF estimates averaged over residual elements and trajectories showed that atom fluctuation within the structures was significantly higher in the second third. The inquiry analyzed the radius of gyration and SASA to determine how the unfolding of the mutant protein structure affects the solubility of surface area and protein activity. A discrepancy in RG values suggests decreased stability. The current results indicate the original structure had a more limited range of RG values than the mutated structures. The solvent-accessible surface area of the wild-type INS differed from that of the mutations of the INS protein. These three mutations, C96Y, P52R, and C96R, are suggested to cause a decrease in *INS* stability and functionality. Figure 7 displays the three-dimensional structure of the selected INS gene mutations, that are likely suggested to introduce structural disruptions potentially affecting the stability and function of the protein.

From the literature, Laurenzano et al., in 2019, detected a P9R missense variant of the INS gene in a patient, which occurs in the signal peptide of the preproinsulin molecule in an amino acid residue that is highly conserved [32]. Also, the results of Balboa et al., in 2018, demonstrated that neonatal diabetes-associated INS mutations lead to defective beta-cell mass expansion, leading to diabetes development [9]. Moreover, Liu et al., in 2015, reported that a preproinsulin-L13R mutation was identified experimentally in a baby girl who developed severe diabetes on the second day after birth [8]. Our results differ from other results in the literature. Hence, we compare the results of our study with the results of other published studies on INS gene mutations associated with PNDM in Table 6.

However, additional research may be required to link these computational results with experimental data to understand the effects of these mutations on protein behavior fully.

## 5. Conclusions

This study delved into the assessment of the potential correlation between non-synonymous single-nucleotide polymorphisms (nsSNPs) within the *INS* gene and PNDM utilizing an array of computational methodologies. Techniques such as SIFT, PolyPhen2, Mutpred2, SNAP2, SNP & Go, PhD-SNP, MuPro, I-Mutant, HOPE, and MDSs were employed. Among the findings, three mutations, C96Y, P52R, and C96R, were identified to diminish the stability and functionality of the INS protein. These results suggest that these mutations could impact the stability and function of the insulin protein encoded by the *INS* gene, potentially influencing the onset of PNDM. Further investigations are warranted to comprehensively grasp the diverse ramifications of *INS* gene mutations on insulin production and function. Upon validation, these findings could be instrumental in genetic screenings for PNDM, aiding in risk assessment and the formulation of therapeutic or preventive measures.

## Figures and Tables

**Table 1:** Functional analysis results using SIFT, PolyPhen-2, and SNAP2 tools. Transcription ID is ENST00000381330.5.

NCBI DATABASE				SIFT		PolyPhen-2		SNAP2	
Variant ID	Allele	MAF	Mutation	Prediction	Score	Prediction	Score	Prediction	Accuracy %
rs28933985	C|G	T = 0./0	R89P	deleterious	0.00	Probably	1.00	74/85	85%
rs28933985	C|T	T = 0./0	R89H	deleterious	0.00	Probably	1.00	68/80	80%
rs80356664	C|G	T = 0./0	G32R	deleterious	0.00	Probably	1.00	95/95	95%
rs80356664	C|T	T = 0./0	G32S	deleterious	0.00	Probably	1.00	88//91	91%
rs80356666	A|C	C = 0./0	C43G	deleterious	0.00	Probably	1.00	86/91	91%
rs80356667	C|A	A = 0./0	G47V	deleterious	0.00	Probably	1.00	85/91	91%
rs80356668	A|C	C = 0./0	F48C	deleterious	0.00	Probably	1.00	81/91	91%
rs80356668	A|G	C = 0./0	F48S	deleterious	0.00	Probably	1.00	89/91	91%
rs80356669	G|A	NA	R89C	deleterious	0.00	Probably	1.00	62/80	80%
rs80356670	C|A	A = 0./0	G90C	deleterious	0.00	Probably	1.00	65//80	80%
rs80356671	C|G	T = 0./0	C96S	deleterious	0.01	Probably	0.995	81/91	91%
rs80356671	C|T	T = 0./0	C96Y	deleterious	0.00	Probably	1.00	87/91	91%
rs80356672	T|C	C = 0./0	Y108C	deleterious	0.00	Probably	1.00	78/85	85%
rs121908261	G|A	A = 0./0	R55C	deleterious	0.00	Probably	1.00	61/80	80%
rs121908272	G|C	NA	H29D	deleterious	0.01	Probably	1.00	78/85	85%
rs121908273	A|G	T = 0./0	L35P	deleterious	0.00	Probably	1.00	96/95	95%
rs121908273	A|T	T = 0./0	L35Q	deleterious	0.00	Probably	1.00	91/95	95%
rs121908276	G|C	NA	S101C	deleterious	0.00	Probably	1.00	54/75	75%
rs121908277	T|C	NA	Y103C	deleterious	0.02	Probably	1.00	55/75	75%
rs121918102	C|A	NA	V92L	deleterious	0.00	Probably	1.00	77/85	85%
rs145038693	G|A	C = 0./0	P52L	deleterious	0.00	Probably	1.00	73/85	85%
rs145038693	G|C	C = 0./0	P52R	deleterious	0.00	Probably	0.998	77/85	85%
rs145038693	G|T	C = 0./0	P52H	deleterious	0.00	Probably	1.00	75/85	85%
rs148685531	G|C	A = 0.000142/2	F49L	deleterious	0.00	Probably	0.994	81/91	91%
rs397515521	C|A	NA	M1I	deleterious	0.00	Probably	0.981	77/85	85%
rs397515521	C|T	NA	M1I	deleterious	0.00	Probably	0.981	77/85	85%
rs765512575	C|T	T = 0.000105/2	G44R	deleterious	0.00	Probably	0.996	86/91	91%
rs1057524907	T|C	NA	E93G	deleterious	0.01	Probably	0.979	77/85	85%
rs1252051752	T|A	NA	S98C	deleterious	0.04	Probably	0.996	37/66	66%
rs1278232284	G|A	A = 0.000004/1	L35V	deleterious	0.00	Probably	1.00	75/85	85%
rs1278232284	G|C	A = 0.000004/1	L35M	deleterious	0.00	Probably	1.00	77/85	85%
rs1564912274	T|C	C = 0.000004/1	H34R	deleterious	0.00	Probably	0.997	64/80	80%
rs1564912274	T|G	C = 0.000004/1	H34P	deleterious	0.00	Probably	0.999	82/91	91%
rs1845839718	A|G	NA	C96R	deleterious	0.00	Probably	1.00	90/95	95%
rs2133672742	C|A	NA	C109F	deleterious	0.00	Probably	1.00	83//91	91%
rs2133672778	A|C	NA	Y108D	deleterious	0.00	Probably	1.00	90/95	95%
rs2133676660	G|A	NA	L39F	deleterious	0.00	Probably	1.00	72/85	85%
rs2133676747	C|A	NA	G32V	deleterious	0.00	Probably	1.00	88/91	91%
rs2133676771	A|C	NA	C31G	deleterious	0.00	Probably	1.00	90//95	95%
rs28933985	C|T	T = 0./0	R89H	deleterious	0.00	Probably	1.00	68/80	80%
rs80356664	C|G	T = 0./0	G32R	deleterious	0.00	Probably	1.00	95/95	95%
rs80356664	C|T	T = 0./0	G32S	deleterious	0.00	Probably	1.00	88/91	91%
rs80356666	A|C	C = 0./0	C43G	deleterious	0.00	Probably	1.00	86//91	91%
rs80356667	C|A	A = 0./0	G47V	deleterious	0.00	Probably	1.00	85/91	91%
rs80356668	A|C	C = 0./0	F48C	deleterious	0.00	Probably	1.00	78/85	85%
rs80356668	A|G	C = 0./0	F48S	deleterious	0.00	Probably	1.00	77/85	85%
rs80356669	G|A	NA	R89C	deleterious	0.00	Probably	1.00	62/80	80%
rs80356670	C|A	A = 0./0	G90C	deleterious	0.00	Probably	1.00	65/80	80%
rs80356671	C|G	T = 0./0	C96S	deleterious	0.01	Probably	0.995	81/91	91%
rs80356672	T|C	C = 0./0	Y108C	deleterious	0.00	Probably	1.00	78/85	85%
rs11557614	G|A	A = 0./0	A38V	deleterious	0.01	Probably	0.991	70/85	85%
rs144093133	C|A	T = 0.000061/6	G75V	deleterious	0.00	Probably	0.999	38/66	66%
rs202244834	T|G	G = 0./0	K53T	deleterious	0.02	Probably	0.963	54/75	75%
rs760425445	C|T	T = 0.000004/1	G90D	deleterious	0.00	Probably	0.987	80/91	91%
rs781016664	G|A	A = 0.000012/3	R56W	deleterious	0.00	Probably	1.00	76/85	85%
rs983508038	C|T	T = 0.000004/1	R56Q	deleterious	0.00	Probably	1.00	57/75	75%
rs1182567488	T|G	G = 0.000071/1	Q28H	deleterious	0.00	Probably	0.995	58/75	75%
rs1184417816	C|T	T = 0.000111/1	R55H	deleterious	0.02	Probably	1.00	60/80	80%
rs1213888316	A|G	G = 0./0	L11P	deleterious	0.00	Probably	0.994	61/80	80%
rs1313322794	C|G	G = 0.000004/1	G47R	deleterious	0.00	Probably	1.00	91/95	95%
rs1313490068	A|G	G = 0.000224/1	L13P	deleterious	0.00	Probably	0.997	83/91	91%
rs1460766978	G|A	A = 0./0	P52S	deleterious	0.00	Probably	1.00	64/80	80%
rs1460766978	G|T	A = 0./0	P52T	deleterious	0.00	Probably	1.00	67/80	80%
rs1845838687	T|C	C = 0./0	Q104R	deleterious	0.02	Probably	0.976	59/75	75%
rs1845873763	C|T	T = 0.00005/1	E59K	deleterious	0.02	Probably	0.986	35/66	66%
rs1845879949	A|T	T = 0./0	L14Q	deleterious	0.00	Probably	1.00	61/80	80%
rs2133677126	A|T	T = 0.00007/2	M1K	deleterious	0.00	Probably	0.995	94/95	95%

NA = not available.

**Table 2:** Pathogenicity analysis results using SNP & GO and PhD-SNP tools.

NCBI DATABASE			SNPs & GO		PhD-SNP.	
Variant ID	Allele	Mutation	Prediction	SCORE	Prediction	SCORE
rs28933985	C|G	R89P	DISESASE	10	DISESASE	2
rs80356671	C|T	C96Y	DISESASE	10	DISESASE	1
rs121908273	A|G	L35P	DISESASE	10	DISESASE	3
rs145038693	G|A	P52L	DISESASE	10	DISESASE	1
rs145038693	G|C	P52R	DISESASE	10	DISESASE	2
rs145038693	G|T	P52H	DISESASE	9	DISESASE	1
rs765512575	C|T	G44R	DISESASE	10	DISESASE	0
rs1564912274	T|C	H34R	DISESASE	10	DISESASE	2
rs1564912274	T|G	H34P	DISESASE	10	DISESASE	1
rs1845839718	A|G	C96R	DISESASE	10	DISESASE	5
rs1460766978	G|A	P52S	DISESASE	10	DISESASE	0
rs1845879949	A|T	L14Q	DISESASE	9	DISESASE	5

**Table 3:** Pathogenicity analysis results using MutPred2 tools.

NCBI DATABASE			MUTPRED2	
Variant ID	Allele	Mutation	EFFECT	SCORE
rs28933985	C|G	R89P	Affect	0.791
rs80356671	C|T	C96Y	Affect	0.860
rs121908273	A|G	L35P	Affect	0.925
rs145038693	G|C	P52R	Affect	0.570
rs765512575	C|T	G44R	Affect	0.716
rs1564912274	T|G	H34P	Affect	0.663
rs1845839718	A|G	C96R	Affect	0.910
rs1845879949	A|T	L14Q	Affect	0.627

**Table 4:** Results of the impact of SNPs on protein stability analysis using I-MUTANT and MuPro tool.

NCBI DATABASE			I-MUTANT		MuPro	
Variant ID	Allele	Mutation	Stability	Reliability Index	EFFECT	Confidence Score
rs28933985	C|G	R89P	DECREASE	5	Increase	0.078515012
rs80356671	C|T	C96Y	DECREASE	4	Decrease	−0.080856117
rs121908273	A|G	L35P	DECREASE	4	Increase	0.065423903
rs145038693	G|C	P52R	DECREASE	0	Decrease	−1.0
rs765512575	C|T	G44R	DECREASE	1	Increase	0.49104088
rs1845839718	A|G	C96R	DECREASE	8	Decrease	−0.574683230494305
rs1845879949	A|T	L14Q	DECREASE	8	Decrease	−0.62610731178958

**Table 5:** Summary of HOPE results for the different amino acid mutations of the *INS* gene.

Variant ID	rs145038693	rs80356671	rs1845839718	rs1845879949
Mutations	P52R	C96Y	C96R	L14Q
	Proline into Arginine at position 52	Cysteine into Tyrosine at position 96	Cysteine into Arginine at position 96	Leucine into Glutamine at position 14
Allele	G|C	C|T	A|G	A|T
Amino Acids Properties				
Size	The mutant residue is bigger than the wild-type residue.	The mutant residue is bigger than the wild-type residue.	The mutant residue is bigger than the wild-type residue	The mutant residue is bigger than the wild-type residue.
Hydrophobicity value	Wild-type residue is more hydrophobic than the mutant residue.	Wild-type residue is more hydrophobic than the mutant residue.	Wild-type residue is more hydrophobic than the mutant residue	Wild-type residue is more hydrophobic than the mutant residue.
Charge	Wild-type residue charge was NEUTRAL	No change	Wild-type residue charge was NEUTRAL	No change
	Mutant residue charge was POSITIVE	---	Mutant residue charge was POSITIVE.	----
Variant’s score	0.90322566	0.9947578	0.9939898	0.8815106
Conservation	Mutant and wild-type residues were not very similar	Wild-type residue was very conserved	Wild-type residue was very conserved	Wild-type residue was very conserved
		The mutant residue was located near a highly conserved position	The mutant residue was located near a highly conserved position	Mutant residue was located near a highly conserved position
Effect of mutation on protein	Probably damaging to the protein.	Probably damaging to the protein.	Probably damaging to the protein.	Mutation occurs without damaging the protein

--- = No change (mutant residue charge); ---- = blank (conservation).

**Table 6:** Comparison of the results of our study with the results of other published studies on INS gene mutations associated with PNDM.

	Studies on INS Gene Mutations Associated with PNDM			
	This Study	Laurenzano et al. 2019 [32]	Balboa et al. 2018 [9]	Liu et al. 2015 [8]
Mutations	P52R	P9R	C96R	L13R
	C96R		C109Y	
	C96Y			

## Data Availability

Data concerning SNPs associated with the INS gene, Insulin protein sequence identified by UniProt ID P01308, and INS nsSNPs are available at the NCBI database, via the URL: http://www.ncbi.nlm.nih.gov/snp/ (accessed on 26 September 2023) and UniProt Knowledgebase database.
